# Convalescent Plasma Transfusion for the Treatment of COVID-19—Experience from Poland: A Multicenter Study

**DOI:** 10.3390/jcm10010028

**Published:** 2020-12-24

**Authors:** Anna Moniuszko-Malinowska, Piotr Czupryna, Dorota Zarębska-Michaluk, Krzysztof Tomasiewicz, Sławomir Pancewicz, Marta Rorat, Anna Dworzańska, Katarzyna Sikorska, Beata Bolewska, Beata Lorenc, Andrzej Chciałowski, Dorota Kozielewicz, Barbara Oczko-Grzesik, Anna Szymanek-Pasternak, Bartosz Szetela, Magdalena Figlerowicz, Magdalena Rogalska, Izabela Zaleska, Robert Flisiak

**Affiliations:** 1 Department of Infectious Diseases and Neuroinfections, Medical University of Białystok, 15-085 Białystok, Poland; avalon-5@wp.pl (P.C.); spancewicz@interia.pl (S.P.); 2 Department of Infectious Diseases, Jan Kochanowski University, 25-369 Kielce, Poland; dorota1010@tlen.pl; 3 Department of Infectious Diseases and Hepatology, Medical University of Lublin, 20-059 Lublin, Poland; krzysztoftomasiewicz@umlub.pl (K.T.); annadw8@gmail.com (A.D.); 4 Department of Forensic Medicine, Wrocław Medical University, 50-367 Wrocław, Poland; marta.rorat@gmail.com; 5 First Infectious Diseases Ward, Gromkowski Regional Specialist Hospital in Wrocław, 51-149 Wrocław, Poland; 6 Department of Tropical Medicine and Epidemiology, Medical University of Gdańsk, 80-210 Gdańsk, Poland; ksikorska@gumed.edu.pl; 7 Department of Infectious Diseases, University of Medical Sciences, 61-701 Poznań, Poland; bbolewska@ump.edu.pl; 8 Department of Infectious Diseases, Medical University of Gdańsk, 80-210 Gdańsk, Poland; lormar@gumed.edu.pl; 9 Department of Infectious Diseases and Allergology, Military Institute of Medicine, 04-141 Warsaw, Poland; achcialowski@wim.mil.pl; 10 Department of Infectious Diseases and Hepatology, Faculty of Medicine, Collegium Medicum in Bydgoszcz, Nicolaus Copernicus University, 87-100 Torun, Poland; d.kozielewicz@wsoz.pl; 11 Department of Infectious Diseases in Bytom, Medical University of Silesia, 40-055 Katowice, Poland; bgrzesik@hoga.pl; 12 Department of Infectious Diseases and Hepatology, Wrocław Medical University, 50-367 Wrocław, Poland; aszymanek7@gmail.com; 13 Department of Infectious Diseases, Liver Diseases and Acquired Immune Deficiencies, Wrocław Medical University, 50-367 Wrocław, Poland; bartoszetela@gmail.com; 14 Department of Infectious Diseases and Child Neurology, Poznan University of Medical Sciences, 61-701 Poznań, Poland; mfiglerowicz@gmail.com; 15 Department of Infectious Diseases and Hepatology, Medical University of Białystok, 15-089 Białystok, Poland; pmagdar@gmail.com (M.R.); robert.flisiak1@gmail.com (R.F.); 16 Department of Pediatrics and Infectious Diseases, Wrocław Medical University, 50-367 Wrocław, Poland; izabela.zaleska9@gmail.com

**Keywords:** convalescent plasma, COVID-19, transfusion

## Abstract

Because the optimal treatment for COVID-19 is still unknown, it is important to explore every potential way of improving the chances of survival for COVID-19 patients. The aim of the study was to analyze the effectiveness of convalescent plasma on COVID-19 patients. The study population consisted of 78 patients diagnosed with COVID-19, selected from the SARSTer national database, who received convalescent plasma. The impact on clinical and laboratory parameters was assessed. A clinical improvement was observed in 62 (79%) patients, and 10 (13%) patients died from COVID-19. No side effects of the convalescent plasma treatment were observed. When plasma was administered earlier than 7 days from diagnosis, the total hospitalization time was shorter (*p* < 0.05). Plasma efficacy was inferior to remdesivir in endpoints such as the necessity and duration of oxygen therapy, the duration of hospitalization, and mortality rate, and inferior to other drugs in the case of the duration of hospitalization and the necessity of constant oxygen therapy, but comparable in most other measured endpoints. A comparison of a 30-day mortality rate in patients who received plasma and remdesivir (4/25, 16%) and who received only plasma (6/53, 11%) showed no significant difference. Convalescent plasma efficacy is inferior to remdesivir when treating COVID-19 patients but the addition of remdesivir to plasma does not improve the treatment effectiveness. In most endpoints, plasma was comparable to other treatment options. In our opinion, convalescent plasma may be used as a supportive treatment in COVID-19 patients because of the low frequency of adverse effects and availability, but must be given as early from the diagnosis as possible.

## 1. Introduction

Passive immunization for the prevention and treatment of human infectious diseases can be traced back to the 20th century. Convalescent blood products, obtained by collecting whole blood or plasma from a patient who has survived a previous infection and developed humoral immunity against the pathogen responsible for the disease and is a possible source of specific antibodies of human origin, could be a valid option in the treatment/prophylaxis of several infectious diseases, both in association with other drugs/preventive measures, and as the only therapy when a specific treatment is not available [1]. Plasma has been used in previous epidemics, for example, the Ebola outbreak [2].

Convalescent plasma transfusion acts in two ways: direct and indirect. The direct mechanisms mainly include complement-dependent cytotoxicity, antibody-dependent cellular phagocytosis, and antibody-dependent cell-mediated cytotoxicity, by which convalescent plasma can eliminate infected cells displaying viral antigens at their surface, in addition to complement-mediated inactivation of viral particles, and/or their phagocytosis.

The indirect mechanisms include the induction of the endogenous antiviral immune responses by convalescent plasma, such as the formation of immune complexes with virions and/or infected cells. It can enhance antiviral cytotoxic T lymphocyte responses through Fc gamma receptors—FcγR-mediated binding to dendritic cells, inhibit Treg expansion and immunosuppressive activity, and induce neutrophils antiviral effects [3].

Apheresis plasma is available in relatively large amounts during pandemics. There is a possibility of frequent donations, while the impact on the donor’s hemoglobin may be reduced due to the reinfusion of the donor’s red blood cells. Moreover, the recruitment of donors living in areas in which an epidemic has broken out can offer the added value of providing specific, artificially acquired passive immunity against the local infectious agent. The limitation of plasma availability is due to the fact that the identification, selection, and recruitment of potential donors can be difficult, as convalescent subjects have to meet donor selection criteria, in compliance with national and World Health Organization (WHO) policies and routine procedures [4].

Based on the beneficial role of plasma in previous epidemics, it may be also potentially useful in the current severe acute respiratory syndrome coronavirus 2-SARS-CoV-2 pandemics. As the optimal treatment for COVID-19 is still unknown and guidelines are constantly changing, it is very important to thoroughly explore every potential way of improving the chances of survival for COVID-19 patients.

The aim of the current study was to analyze the effectiveness of convalescent serum treatment on COVID-19 patients in Poland.

## 2. Experimental Section

### 2.1. Materials

The study is based on the SARSTer national database. This ongoing project, supported by the Polish Association of Epidemiologists and Infectiologists, is a national real-world experience study assessing treatments in patients with COVID-19. Patients whose data are collected in the SARSTer database were treated in 30 Polish centers between 1 March and 30 October 2020. The decision about the treatment regimen was made entirely by the treating physician with respect to the current knowledge and recommendations of the Polish Association of Epidemiologists and Infectiologists [5,6].

In the current study, we aimed at assessing plasma treatment effectiveness. A total of 78 patients (32 female, 46 male) diagnosed with COVID-19, with a mean age of 59.5 ± 18.8 years, who received convalescent plasma, were selected from the whole SARSTer database. These were all the patients treated with plasma in the whole database, hospitalized between June 12 and October 30, 2020, in infectious diseases wards located in 16 medical centers in Poland.

The mean body mass index (BMI) of included patients was 28 ± 5.38 kg/m^2^. A total of 31 (39.7%) patients had hypertension, 16 (20.5%) had diabetes, 2 (2.4%) had chronic obstructive pulmonary disease, 12 (15.4%) had coronary disease, 5 (6.4%) had neoplasms, and 1 (1.2%) had AIDS. There were single instances of the following diseases: chronic kidney disease, rheumatoid arthritis, and gout.

All the patients received at least one dose of convalescent plasma (200–267 mL), and 24 (30.7%) patients were given a second dose. The mean time from the onset of symptoms to plasma administration was 6.6 ± 9.7 days. Plasma donors were qualified according to Polish guidelines, which are compliant with the European ones [7].

The SARSTer study was approved by the Ethical Committee of the Medical University of Białystok. In each site, the local bioethics committees approved the treatment.

Patients included in the study met the following criteria: a cough, dyspnea, or fever (>38 °C); a positive result of a polymerase chain reaction (PCR) test for SARS-CoV-2 from a nasopharyngeal swab (the diagnosis of a SARS-CoV-2 infection was confirmed by a reverse transcription-polymerase chain reaction (RT-PCR) from nasopharyngeal or oropharyngeal swabs); typical lesions visible on a chest x-ray (air-space opacification) or chest computerized tomography computer tomography (CT); ground glass; thickened interlobular and interlobular lines in combination with a ground glass pattern—crazy paving; consolidation, widening of the vessels); a need for continuous oxygen therapy; and an oxygen saturation of ≤94% at any time after admission.

For comparative purposes, we built two control groups (also picked from the SARSTer database). The first group (CG I) consisted of 236 patients (93 females and 143 males, with a mean age of 58.6 ± 14.4 years). The second group (CG II) consisted of 715 patients treated with other drugs (not plasma or remdesivir) (335 females and 380 males, with a mean age of 52.2 ± 21.5 years). In this group, 43/715 (6%) patients received tocilizumab, 70/715 (9.7%) patients received dexamethasone, 313/715 (43.7%) received chloroquine, 63/715 (8.8%) received hydroxychloroquine, 202/715 (28.2%) patients received lopinavir/ritonavir, 260/715 (36%) received azithromycin, and 312/715 (43.6%) received fractioned heparin.

We decided to subtract remdesivir as a separate group because so far it is the only drug with proven antiviral efficacy and it should be given at the same phase of the disease as plasma.

Additionally, for this comparison, from the plasma group, we picked only patients who received plasma during the first seven days from the onset of the disease (Plasma I Group), as plasma is the most effective during the early phase of the disease. This group consisted of 55 patients: 20 females, 35 males, with a mean age of 59.9 ± 18.2 years.

### 2.2. Methods

Clinical and laboratory parameters were compared. Laboratory parameters were analyzed at five time points: at admission, 0–2 days after treatment, 3–5 days after treatment, 6–10 days after treatment, and >10 days after treatment.

Depending on the severity of the disease, the patients were divided into 8 categories on an ordinal scale:

1: Not hospitalized, normal activity

2: Not hospitalized, with impaired activity and/or requiring oxygen support

3: Hospitalized, not requiring oxygen support or medical care

4: Hospitalized, not requiring oxygen support but requiring medical care (connected or not connected with COVID-19)

5: Hospitalized, requiring oxygen support

6: Hospitalized, requiring noninvasive high-flow oxygen support (high-flow nasal cannula)

7: Hospitalized, requiring mechanical ventilation or extracorporeal membrane oxygenation (ECMO)

8: Death

The major endpoints assessed in the study were the following:

- The necessity of constant oxygen therapy (% of patients)
- The duration of oxygen therapy (days)
- The necessity of artificial ventilation (% of patients)
- The duration of hospitalization (days)
- Mortality rate (% of patients)
- Clinical improvement, defined as moving down 2 points on the ordinary clinical scale, measured at four time points (7th day, 14th day, 21st day, and 28th day)

### 2.3. Statistical Analysis

Data were presented as means and standard deviations or medians (interquartile ranges), as appropriate. Groups were compared using the Mann-Whitney U test and the Chi-square test. To compare the frequencies of the variables before and after treatment with convalescent plasma, a paired Wilcoxon signed-rank test was used. The survival rate was presented as Kaplan-Meier curves. Kaplan-Meier curves were compared with a log-rank test. A *p* < 0.05 was considered significant. The Statistica 13 software(StatSoft Polska Sp. Z o.o., Cracov, Poland) was used.

## 3. Results

### 3.1. Plasma Group Results (Whole Group)

At admission, 56 (71.9%) patients complained of a cough and fever—68 (87.2%), dyspnea—37(47.43%), changed smell and/or taste—11 (14.1%), diarrhea—12 (15.4%), headaches—6 (7.7%), nausea—4 (5%), vomiting—3 (3.8%), and general weakness—30 (38.5%). The total hospitalization times were 21.8 ± 11.4 days. Typical lesions were observed in chest CTs of 39 (50%) patients.

Results of laboratory tests before admission and after treatment are presented in Table 1. Statistically significant differences between SpO_2_, C Reactive Protein (CRP) concentration, white blood cells (WBC), lymphocytes % and platelets (PLT) number, alanine aminotransferase (ALT) activity, and interleukin-6 (IL-6) concentration at admission and after the treatment were observed (*p* < 0.05).

The mean oxygen saturation before treatment was 89% ± 5.4%, while after the convalescent plasma treatment, it was 96.78 ± 2%. The mean duration of oxygen therapy was 13.46 ± 10.8 days. Nine (11.5%) patients required mechanical ventilation.

The comparison of obese (BMI > 30) and normal-weight patients treated with convalescent plasma did not show any significant differences as far as the outcome of the disease was concerned.

**Figure 1:**
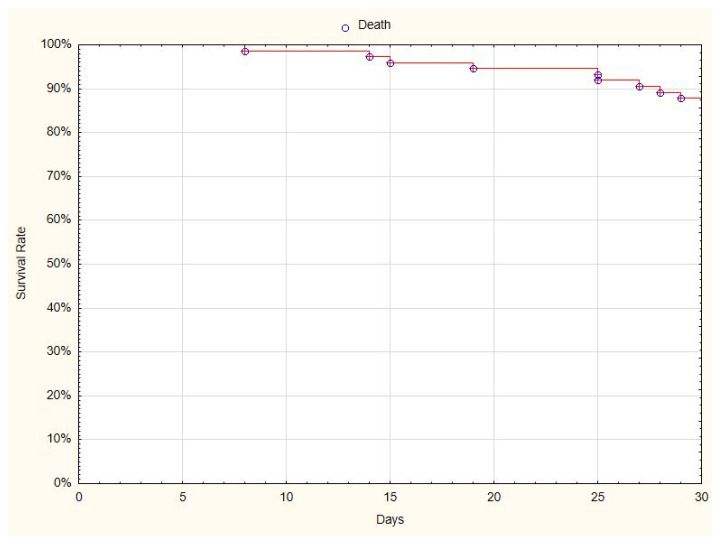
Kaplan-Meier curve presenting the 30-day survival rate of patients treated with convalescent plasma.

The patients were scored on a clinical scale at admission, and 7, 14, 21, and 28 days after admission. At the end of observation, 68 (87%) patients had recovered from COVID-19 (Figure 1), and 10 (13%) patients had died by the 30th day of observation.

Clinical improvement, defined as moving down by 2 points on the ordinary clinical scale before and after treatment, was observed in 62 (79%) patients.

No side effects of convalescent plasma treatment were observed in our patients.

### 3.2. Comparison of Results Obtained From Two Subgroups of Patients: Plasma I Group vs. Plasma II Group

As it is known that immunoglobulins should be administered during the viremic phase of the disease, we compared the results obtained from two subgroups of patients: those who received plasma in the first seven days after the onset of the disease and those who received plasma more than seven days after the onset of the disease (Table 2). When plasma was administered earlier than seven days from the diagnosis, the total hospitalization time was shorter (18.96 ± 7.05 vs. 28.39 ± 16.18 days; *p* < 0.05). The duration of oxygen therapy did not differ between both groups (11.31 ± 6.61 vs. 21.36 ± 18.23 days; *p*-not significant).

The comparison of the number of patients with clinical improvement, defined as moving down on the clinical scale by 2 ranks at four time points, in the group of patients who received plasma in the first seven days after the onset of the disease and those who received plasma more than seven days after the onset of the disease, was performed. Clinical improvement was observed in 39 out of 55 (70%) patients who received plasma in the first seven days after the onset of the disease, versus 9 out of 23 (39%) patients who received plasma more than seven days from the onset of the disease on Day 21 (*p* < 0.05). It was also seen in 48 out of 55 (87%) patients who received plasma in the first seven days after the onset of the disease, versus 14 out of 23 (60.9%) patients who received plasma later than seven days after the diagnosis on Day 28 (*p* < 0.05). No differences were observed between the age groups (Table 2).

**Figure 2:**
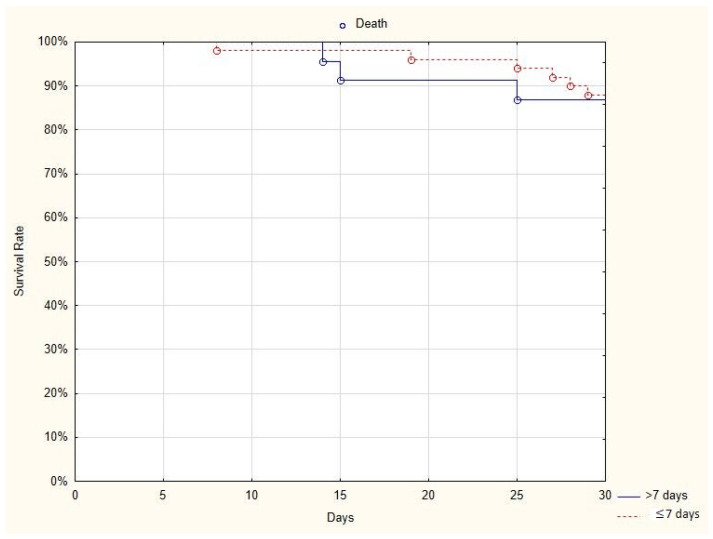
Kaplan-Meier curve presenting the 30-day survival rate of patients who received plasma in the first seven days after the onset of the disease and those who received plasma more than seven days after the onset of the disease.

The comparison of the 30-day mortality rate in two subgroups of patients—those who received plasma in the first seven days after the onset of the disease (6/55, 11%) and those who received plasma more than seven days after the onset of the disease (4/23, 17%)— showed no difference between groups (log-rank *p* = 0.86) (Figure 2).

### 3.3. Comparison of the Results Obtained From Two Subgroups of Patients: Plasma and Remdesivir vs. Only Plasma

**Figure 3:**
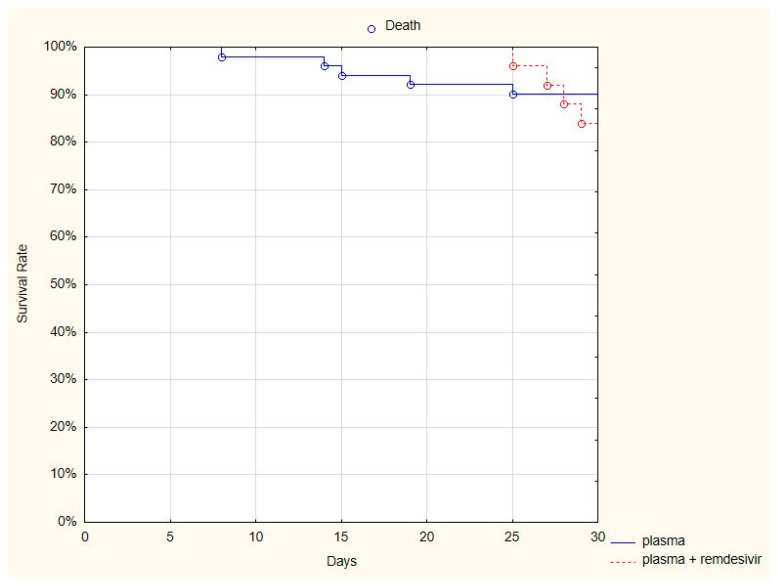
Kaplan-Meier curve presenting the 30-day survival rate of patients treated with convalescent plasma and remdesivir, and only plasma.

Remdesivir is the only registered antiviral regimen; therefore, we compared whether the addition of remdesivir improved the survival rate of patients treated with both antiviral and plasma versus only plasma. A comparison of the 30-day mortality rate in two subgroups of patients—those who received both the plasma and remdesivir (4/25, 16%) and those who received only plasma (6/53, 11%)—showed no difference between groups (log-rank *p* = 0.66) (Figure 3).

Furthermore, no differences were observed as far as the duration of hospitalization, the necessity of mechanical ventilation, or the duration of oxygen therapy were concerned. The necessity of permanent oxygen therapy was more frequent in patients who received both plasma and remdesivir (*p* < 0.05). Patients’ clinical statuses assessed at five time points showed a difference only after seven days (a better outcome was observed in the plasma group, *p* < 0.05).

### 3.4. Comparison of Results Obtained From Two Subgroups of Patients: <60 Years Old and ≥60 Years Old

We divided the Plasma Group into 2 subgroups:

I—patients younger than 60 years old
II—patients ≥60 years old

The results of the laboratory tests and clinical parameters in each subgroup are presented in Table 2 and Table 3. The statistical differences between these groups were stated when comparing oxygen saturation at admission and CRP concentration at admission, but not after treatment. Additionally, the differences were observed when comparing the necessity of oxygen therapy and the duration of oxygen treatment between Groups I and II (8 ± 3.5 vs. 15.3 ± 11.8 days; *p* < 0.05). The duration of hospitalization did not differ between the groups (22.7 ± 12.9 vs. 21.2 ± 10.4 days; *p*-non statistical).

The comparison of the number of patients with clinical improvement, defined as moving down in the clinical scale by 2 ranks at four time points, in Groups I and II, showed no differences between the age groups (Table 3).

### 3.5. Comparison of Patients Who Received Plasma in the First Seven Days after the Onset of the Disease, Patients Who Received Only Remdesivir (CG I), and Patients Who Received Multiple Drugs, Such as Dexamethasone, Lopinavir/Ritonavir, Tocilizumab, Hydroxychlorochine, Chlorochine, Azitromycine (CG II)

The necessity of constant oxygen therapy was less frequent in CG I than in the Plasma Group (41/55 (74.5%) vs. 108/235 (46%); *p* < 0.05). In addition, the duration of oxygen therapy and the duration of hospitalization was shorter in CG I (11.3 ± 6.6 vs. 8.3 ± 8.6 days; *p* < 0.05 and 19 ± 7.1 vs. 14.4 ± 7.5 days; *p* < 0.05, respectively). The mortality rate was significantly lower in CG I (8/235 (3.4%) vs. 6/55 (11.2%); *p* < 0.05).

The necessity of mechanical ventilation was more frequent in patients treated with plasma than those treated with remdesivir (6/55 (11.2%) vs. 10/235 (4%)), but the results were at the edge of statistical significance—*p* = 0.05)).

The comparison between the Plasma Group and CG II showed that the necessity of constant oxygen therapy was less frequent in CG II (41/55 (74.5%) vs. 276/715 (38.6%); *p* < 0.05). Furthermore, the duration of hospitalization was shorter in CG II (19 ± 7.1 vs. 15.7 ± 10.4 days; *p* < 0.05).

The duration of oxygen therapy in the Plasma Group and CG II was 11.3 ± 6.6 vs. 10.2 ± 8.5 days, the necessity of mechanical ventilation in the Plasma Group and CG II was 6/55 (11.2%) vs. 30/715 (4.2%), and the mortality rate in the Plasma Group and CG II was 6/55 (11.2%) vs. 43/715 (6%), but the differences were not statistically significant.

The comparison of the number of patients with clinical improvement, defined as moving down in the clinical scale by 2 ranks at four time points, showed no statistically significant difference at any time point between Plasma I Group and both CG I and CG II (Table 4).

## 4. Discussion

The results of our study suggest that convalescent plasma may be considered as a supportive treatment for COVID-19 patients, although more clinical trials are necessary to compare the effect with other therapies (e.g., remdesivir).

Until now, only a few randomized studies on the effectiveness of convalescent plasma therapy have been published. In the study of Li et al., which included 103 patients with severe or life-threatening COVID-19, convalescent plasma therapy that was added to standard treatment, compared with standard treatment alone, did not result in a statistically significant improvement in time to clinical improvement within 28 days [8].

Rajendran et al., at the beginning of the COVID-19 pandemic, performed a review based on 5 out of 110 records identified through a database search from PubMed, EMBASE, and Medline databases, and concluded that convalescent plasma may reduce mortality in critically ill patients, influence the disappearance of SARS-CoV-2 RNA, and the release of clinical symptoms. Based on the limited scientific data, convalescent plasma therapy in COVID-19 patients appears to be safe, clinically effective, and reduces mortality [9]. The results and conclusions of this study are in contrast to recent observations and guidelines that do not recommend the use of convalescent plasma in critically ill patients.

Abolghasemia et al. reported that only 8 patients out of 115 (7%) treated with convalescent plasma required intubation, in opposition to 20% of patients from the control group [10]. No significant differences in the mortality rate between the groups were observed (14.8% vs. 24.3%). In our study, 11.5% of patients required intubation and artificial ventilation. The mortality rate in our group was 13%, which is similar to the observations of Joyner et al., who observed 20,000 patients and a mortality rate of 13% [11].

In contrast to the observation of Zheng et al., six COVID-19 subjects with respiratory failure received convalescent plasma at a median of 21.5 days after the first detection of viral shedding, all tested negative for SARS-CoV-2 RNA by three days after infusion, and five eventually died. It may be concluded that the time when plasma is given plays an important role, and treatment should be initiated earlier, not in critical, end-stage COVID-19 patients [12]. In our study, the mean time from the beginning of the disease to plasma administration was 6.6 ± 9.7 days. Additionally, the comparison of the results between the patients who received plasma in the first seven days from the onset of the disease and those who received plasma more than seven days from the onset of the disease showed differences on the clinical scale after 21 and 28 days, and in the duration of hospitalization. Data from the United States shows that plasma is most effective if used in the first three days of hospitalization [13,14]. In our study, we divided the groups into >7 days and >7 days. We assumed seven days was accurate because patients were admitted to the hospital late from the onset of the disease, and seven days is the time period that the virus replicates; thus, following the pathomechanism of the disease it seemed reasonable to include patients with <7 days in the analysis.

When comparing the laboratory test results, we observed the differences between SpO_2_, CRP concentration, WBC, lymphocytes % and PLT number, ALT activity, and IL-6 concentration at admission and after the treatment. These were in accordance with the observations of Duan et al., who noticed that several parameters tended to improve, as compared with pretransfusion, including decreased C-reactive proteins (55.98 mg/L vs. 18.13 mg/L) [15].

As obesity is one potential risk factor for an unfavorable course of the disease, we divided the patients into two groups: BMI < 30 kg/m^2^ and ≥30 kg/m^2^; however, we did not notice any differences.

As age is also an important risk factor, we compared two groups of patients—those under 60 years old and those over 60 years old. At admission, the elder group was, in general, in worse condition, and SpO_2_ was lower than in younger patients. Moreover, they required longer oxygen therapy, but the final hospitalization duration did not differ, and the results obtained after treatment did not differ from the younger patients. It can be concluded that older patients may benefit from convalescent plasma donation, as it also seems to have fewer adverse effects than other potential therapies.

It should be noted that, although plasma transfusions carry a risk of side effects such as severe allergic transfusion reactions, transfusion-associated circulatory overload, and transfusion-related acute lung injury, none of these kinds of situations were observed in our study or in the study of Duan et al. In the observations of Joyner et al., of 5000 patients, only 2 of 36 serious adverse events were judged as definitely related to the convalescent plasma transfusion by the treating physician, thus, the authors concluded that these early indicators suggest that transfusion of convalescent plasma is safe in hospitalized patients with COVID-19 [16].

In late October 2020, the results of a large Indian cohort PLACID study were published. In the study, 235 adult patients received two doses of 200 mL convalescent plasma, transfused 24 h apart. Progression to severe disease or all-cause mortality at 28 days after enrolment occurred in 19% of patients in the intervention arm and 18% in the control arm, thus, the authors concluded that therapy with convalescent plasma was not associated with a reduction in progression to severe COVID-19 or all-cause mortality [17].

In our study, the plasma efficacy was inferior to remdesivir in some major endpoints, such as the necessity and duration of oxygen therapy, the duration of hospitalization, and the mortality rate. It was also inferior to other drugs in the cases of the duration of hospitalization and the necessity of constant oxygen therapy, but comparable in most of the other measured endpoints.

Furthermore, our study shows that the addition of remdesivir to plasma did not make any difference in most endpoints, such as mortality rate, duration of hospitalization, the necessity of mechanical ventilation, and duration of oxygen therapy. The necessity of permanent oxygen therapy was even more frequent in patients who received both plasma and remdesivir.

Based on these results of our study, we suggest that remdesivir must be favored in the early stages of COVID-19. However, due to a remdesivir supply shortage all over the world, other therapeutic options for COVID-19 are needed.

Even though the results of our study have not proven plasma effectiveness, in our opinion, convalescent plasma may still play a role in the treatment of COVID-19 patients. In most endpoints, the results of patients treated with plasma were comparable to the results of patients treated with multiple drugs (except remdesivir). If we take into consideration that plasma is easily obtainable, and a safe treatment option, it is too early to completely resign from this alternative. It should be considered in national and international guidelines, particularly for patients in the early stages of COVID-19. Probably the most important challenge is to find appropriate patients and donors.

The limitations of our study are similar to most prespecified, intention-to-treat analyses. The biggest limitation of our study was the small number of patients who received plasma, especially in the first days after the onset of the disease. This might affect the results, as plasma seems to be the most effective when given as early as possible. Moreover, the plasma group appears to have been sicker than the other groups. Therefore, the results should be interpreted with care. The second limitation was the lack of information about the level of anti-SARS-CoV-2 antibodies and their neutralizing capacity in plasma given to patients. The results should also be interpreted with caution, due to the differences in clinical practices between hospitals (e.g., with regard to standard treatment, supportive treatment, indications for hospitalization, indications for intubation, and various times from diagnosis to plasma treatment implementation). The same issue was raised in all multi-center studies in COVID-19 patients. Moreover, the results of the treatment can be influenced by many factors, such as the quality of the plasma used, the duration of its administration, and the characteristics of patients enrolled in the study. In addition, the control group was very heterogeneous due to multiple drugs used.

The desperate seek for a COVID-19 treatment may influence the quality of pandemic research data. High-quality clinical research must be an integral part of a coordinated international response. On the other hand, even a low number cohort study may support interventions that are important for COVID-19 patients. Both super optimistic and hyper negative conclusions are not welcome.

Even if we admit the limitations of ours and similar studies, we can conclude the following.

## 5. Conclusions

1. Convalescent plasma efficacy is inferior to remdesivir when treating COVID-19 patients.
2. The addition of remdesivir to plasma does not improve treatment effectiveness.
3. Convalescent plasma may be used as a supportive treatment in COVID-19 patients, but must be given as early as possible from the diagnosis.
4. Convalescent plasma might be considered as a safe alternative for other COVID-19 therapies because of the low frequency of adverse effects.

## Figures and Tables

**Table 1:** Results of laboratory tests before and after treatment.

	At Admission					After Treatment					*p*
	Mean	SD	Median	Q25	Q75	Mean	SD	Median	Q25	Q75	
CRP (mg/L)	90	86	62.7	23	136.4	12.0	18.4	4.3	3	11.5	<0.05
PCT (ng/mL)	0.4	1.3	0.1	0.1	0.2	0.4	0.4	0.1	0	0.8	0.24
WBC (1/µL)	6427.4	3453.3	5900	3900	8740	6545.3	4528.4	5805	4340	7450	0.16
ALT (IU/L)	41.6	37.4	30.2	21	47	57.5	35.7	52.6	32	80	<0.05
PLT (1/µL)	183,517.3	92,510.3	182,500	132,000	212,000	206,663.5	131,822	196,500	111,000	268,000	0.41
Il-6 (pg/mL)	92.8	126.4	46	16.2	105.4	27.3	31.8	14.8	7	39	<0.05
d-dimer (ng/mL)	1874.5	5605	717	483	1170	1376.6	1329.9	834	377	2024.5	0.96

CRP: C Reactive Protein; PCT: procalcitonin; WBC: white blood cells; PLT: platelets; ALT: alanine aminotransferase; IL-6: interleukin-6; Q25/75: interquartile range; SD: standard deviation.

**Table 2:** The comparison of the frequency of clinical improvement, defined as moving down by 2 points on the ordinary clinical scale, between patients who received plasma ≤7 days and >7 days from diagnosis and age groups.

Examination at Day	Plasma Administered ≤7 Days from Diagnosis *n* = 55				Plasma Administered >7 Days from Diagnosis *n* = 23				*p* Overall
	Overall *n* = 55	Age < 60 *n* = 22	Age > 60 *n* = 33	*p*	Overall *n* = 23	Age < 60 *n* = 9	Age > 60 *n* = 14	*p*	
7	3 (5.5%)	1 (4.5%)	2 (6.1%)	ns	1 (4.3%)	0	1 (7.1%)	ns	ns
14	20 (36.4%)	11 (50%)	9 (27.3%)	ns	4 (17.4%)	1 (11.1%)	3 (21.4%)	ns	ns
21	39 (70.1%)	18 (81.9%)	21 (63.6%)	ns	9 (39.1%)	3 (33.3%)	6 (42.9%)	ns	<0.05
28	48 (87.3%)	21 (95.5%)	27 (81.8%)	ns	14 (60.9%)	5 (55.6%)	9 (64.3%)	ns	<0.05

**Table 3:** Results of laboratory tests in patients <60 years old and ≥60 years old.

	<60 Years Old					≥60 Years Old					
	Mean	SD	Median	Q25	Q75	Mean	SD	Median	Q25	Q75	*p*
At admission											
CRP (mg/L)	76.5	96.6	29.3	8.4	123	98.9	78	81.9	37.3	149	<0.05
PCT (ng/mL)	0.3	0.7	0.1	0	0.3	0.5	1.6	0.1	0.1	0.2	NS
WBC (1/µL)	5662.1	3329.7	4460	3450	8060	6932.1	3475.1	6090	4060	9160	NS
ALT (IU/l)	48.5	49.2	36	20	56	36.9	26.4	27.3	21	45	NS
PLT (1/µL)	169,907.4	91,657.1	176,000	132,000	209,000	192,494	92,948.9	184,000	131,000	224,000	NS
Il-6 (pg/mL)	96.2	150.4	31.1	5	128.2	91.5	119.6	48	21.8	83	NS
d-dimer (ng/mL)	2112.8	6836.8	680	470	1170	1722.4	4730.4	760	483	1237	NS
After treatment											
CRP (mg/L)	14.8	26.4	3.6	3	13.2	10.6	14.1	5	3	11.5	NS
PCT (ng/mL)						0.4	0.4	0.3	0.1	0.8	
WBC (1/µL)	4651	3381.3	5450	1645	6595	7387.2	4796.8	6005	4760	7630	NS
ALT (IU/L)	59.1	19.6	55	42	76.7	56.9	52.6	40.7	21.3	80.5	NS
PLT (1/µL)	236,406.4	180,803.5	215,500	97,000	375,500	193,444.4	107,203.6	196,500	111,000	241,000	NS
Il-6 (pg/mL)						17.4	16.1	13.5	3	34	
d-dimer (ng/mL)	378.3	66	365	320	450	1607	1379.7	1040	439	2200	NS

**Table 4:** **The** comparison of the frequency of clinical improvement, defined as moving down by 2 points on the ordinary clinical scale between patients from Plasma I Group, Control Group (CG) I, and CG II.

Examination at Day	Plasma I	CG1	CG2	*p* Plasma vs. CG1	*p* Plasma vs. CG2
7	3/55 (5.4%)	101/715 (14%)	16/236 (6.77%)	NS	NS
14	20/55 (36.36%)	381/715 (53.3%)	131/236 (55.5%)	NS	NS
21	39/55 (70.9%)	551/715 (77.06%)	194/236 (82.2%)	NS	NS
28	48/55 (87.3%)	630/715 (88.11%)	208/236 (88.13%)	NS	NS
